# The Impact of Negative Emotions on Binge Eating and BMI Among Medical Students

**DOI:** 10.7759/cureus.44499

**Published:** 2023-08-31

**Authors:** Khan W Ali, Malek M Alkrad, Sana A Sankari, Shouq Z Alshihab, Abdulrahman M Aloufi, Farah M Alrifai, Abdulwahab A Alshehri

**Affiliations:** 1 Department of Medicine, Almaarefa University, Riyadh, SAU

**Keywords:** academic stress, emotional eating, saudi arabia, depression, body mass index: bmi, fast food consumption, medical school students, binge eating disorder (bed)

## Abstract

Background

Binge eating disorder (BED) is a condition characterized by episodes of consuming large amounts of food and feeling a lack of control over eating behavior. Stress, anxiety, and depression are risk factors for developing BED, which may be particularly relevant for medical students who experience high levels of academic pressure and stress. This study aims to investigate the prevalence of binge eating behaviors among medical students, as well as the types of negative emotions that are most strongly associated with binge eating.

Methodology

This cross-sectional study employed convenience sampling and included 332 medical students from Almaarefa University in Riyadh, Saudi Arabia. All medical students of all academic levels were eligible to participate in this study. Students from other colleges such as pharmacy students or students from other universities were excluded from this study. The research questionnaire collected information about negative emotions, BMI, number of meals, consumption of fast food, overeating behavior, and relevant demographic data. Descriptive and inferential statistics were used to analyze the data using SPSS Statistics version 26 (IBM Corp. Released 2019. IBM SPSS Statistics for Windows, Version 26.0. Armonk, NY: IBM Corp.).

Results

The descriptive analysis showed that the majority of students reported consuming two meals per day and having snacks twice a day. Fast food consumption was reported by 58.1% of students. The study found that negative emotions such as stress, depression, and loneliness were significantly associated with binge eating behavior and had an impact on weight and BMI. The findings suggest the need for interventions to address negative emotions and promote healthy eating habits among medical students.

Conclusion

The study concludes that negative emotions such as stress, aggression, and boredom are significantly associated with binge eating behaviors among medical students. Depression, disappointment, and thoughts about difficult tasks were linked to detrimental effects on BMI and weight loss.

## Introduction

Binge eating disorder (BED) is a condition in which individuals experience episodes of eating large amounts of food and feel a lack of control over their eating behavior [1]. A meta-analysis of European studies conducted in 2015 and the first half of 2016, which investigated the prevalence, incidence, comorbidity, course, consequences, and risk factors of eating disorders, revealed that the past-year prevalence of BED in adults was estimated to be 1.3%, with a higher prevalence of 1.5% in women compared to 0.3% in men [2]. Other studies have identified that minor stressors that occur on a daily basis and keep the stress system constantly activated could potentially affect the brain's reward and motivation pathways, which may result in an increased desire for and consumption of highly palatable foods [3].

Medical students commonly experience a variety of stressors, as it has been reported that the high incidence of anxiety, stress, and depression among medical students is mainly attributed to academic pressure, familial expectations, and fear of entering the medical field [4-6]. Other studies reported that high stress levels and severe concerns about body shape were linked to a higher risk of eating disorders among medical students [7]. A number of studies, using different instruments to measure emotional eating, have also demonstrated an association between binge eating and weight gain in response to negative emotions like depression and anxiety [8]. A meta-analysis of 35 studies with a total of 21,383 medical students found a pooled prevalence rate of 17.35% for symptoms of feeding and eating disorders (FEDs). The inclusion criteria for this meta-analysis were as follows: (1) studies had to be published in the English language; (2) studies had to be published before September 15, 2021; (3) studies had to focus on undergraduate medical students; and (4) studies had to report the prevalence of FED symptoms or risk factors in the target population [9].

The high prevalence of FEDs in medical students highlights the need for better prevention and treatment programs. Studies have shown that early detection during the premedical years may be vital, and prevention programs targeting medical students have shown positive results [10]. The relationship between negative emotions, binge eating, emotion regulation, and BMI in adults is not yet fully understood [11]. Specifically, how negative emotions may contribute to binge eating and how these behaviors in turn may affect an individual's BMI. The investigation of this relationship provides valuable insights into the potential health consequences of this disorder. Additionally, this study could contribute to a better understanding of the psychological and behavioral factors that contribute to binge eating among medical students. This study aims to investigate the prevalence of binge eating behaviors among medical students, as well as the types of negative emotions that are most strongly associated with binge eating.

## Materials and methods

Study group

This cross-sectional study employed convenience sampling and included 332 medical students from Almaarefa University in Riyadh, Saudi Arabia. The participant interviews for this research took place between July 14, 2023, and July 24, 2023. All medical students of all academic levels were eligible to participate in this study. Students from other colleges such as pharmacy students or students from other universities were excluded from this study.

Data collection method

The data collection tool used in this study was a questionnaire that was specifically designed to gather information on negative emotions, such as stress, anxiety, and depression, as well as BMI, number of meals per day, consumption of fast food, overeating, and demographic data. The questionnaire was developed by the researchers for the purpose of this study and has not been previously validated or tested for reliability. A pilot test of the questionnaire was conducted with a small group of participants (n=10) to identify any potential issues with the questions or response options. The face validity of the specially designed questionnaire used in this study was established by a team of experts in the field from Almaarefa University. These experts reviewed the questionnaire to ensure that the questions appeared to measure what they were intended to measure and were relevant to the study objectives. The team of experts evaluated the questionnaire for its clarity, appropriateness, and relevance to the target population. In this study, the questionnaire was self-reported, meaning that participants themselves filled out the questionnaire either in a paper-based format or through an online questionnaire. They were provided with instructions to answer all questions as accurately and honestly as possible. The questionnaire consisted of multiple-choice and open-ended questions. The questionnaire used in this study was administered in English as all the targeted participants, who were medical students in the college, were proficient in English. Therefore, there was no need for translation as English was already understood by the target population. It should be noted that an Arabic version of the questionnaire was also available upon request. This option was provided to accommodate participants who preferred or required the questionnaire in Arabic.

BMI

The WHO BMI classification [12] is a system that categorizes individuals based on their BMI measurements. BMI is calculated by dividing a person's weight in kilograms by the square of their height in meters. For adults over 20 years old, BMI falls into one of the following categories (Table 1).

**Table 1:** BMI

BMI	Nutritional status
Below 18.5	Underweight
18.5-24.9	Normal weight
25.0-29.9	Pre-obesity (overweight)
30.0-34.9	Obesity class I
35.0-39.9	Obesity class II
Above 40	Obesity class III

These categories were used in the research to classify participants' BMI measurements and analyze the distribution of BMI within the sample population.

Ethical consideration

Ethical approval of the study was obtained from the Institutional Review Board of Almaarefa University with the research project number IRB23-062. Written consent was taken from each participant before collecting any data. Additionally, the data was stored securely with professional data collectors and was only accessible to authorized personnel. Identifiable information, such as names and contact information, was not included in this research.

Statistical analysis

The data was cleared, coded, and entered using SPSS Statistics version 26 (IBM Corp. Released 2019. IBM SPSS Statistics for Windows, Version 26.0. Armonk, NY: IBM Corp.) Descriptive statistics were used to summarize the data, including frequencies and percentages. To assess the relationships between variables, inferential statistics was used, including t-tests, chi-square tests, and correlation analyses. The level of statistical significance was set at a p-value of <0.05, indicating that a result is considered statistically significant if the p-value is less than 0.05. Data was transformed to enhance the interpretability of results. Frequency distribution rendered cumulative percentages to delineate the demographic features, the causes of binge eating, and eating habits. ANOVA appraised the relationship of the dependent variable (eating habits) with the independent variables (different negative emotions that were relieved). A two-tailed t-test was employed to analyze the effect of negative emotion-linked eating (NELE) on BMI and weight. The calculation of sample size was based on the following considerations: Z = standard normal distribution value at 95% confidence level = 1.96 and margin of error (d) = 5%.

## Results

Participant’s characteristics

The descriptive analysis of the study variables is summarized in Table 2, which represents 332 participants in total, 60.2% women (N=200) and 39.8% (N=132) men, with an average age of 22.10 years (SD=2.05), height 166.3 cm (SD=9.66), weight 65.98 kg (SD=15.46), and BMI 23.69 kg/m2 (SD=4.47).

**Table 2:** Demographic traits of the study sample (N=332)

Demographic characteristics	Mean ± SD
Age	22.10 ± 2.05
Height	166.3 ± 9.66
Weight	65.98 ± 15.46
BMI	23.69 ± 4.47
Gender (%)	
Male	132 (39.8)
Female	200 (60.2)
Diet intake (Mean ± SD)	
Number of meal intake per day	2.27 ± 0.85
Number of snack intake per day	1.98 ± 1.174
WHO BMI obesity classification	
Normal range (18.5-24.9)	204 (61.4)
Obese Class 1 (30-34.9)	22 (6.6)
Obese Class 2 (35-39.9)	8 (2.4)
Obese Class 3 (≥40.0)	2 (0.6)
Overweight (≥25)	69 (20.8)
Underweight (<18.5)	27 (8.1)
Academic level (%)	
Level 1	7 (2.1)
Level 2	18 (5.4)
Level 3	16 (4.8)
Level 4	35 (10.5)
Level 5	17 (5.1)
Level 6	40 (12.0)
Level 7	20 (6.0)
Level 8	44 (13.3)
Level 9	53 (16)
Level 10	34 (10.2)
Level 11	14 (4.2)
Level 12	22 (6.6)
Level 13	12 (3.6)
Level 14	12 (3.6)
Socioeconomic states	
High	75 (22.6)
Low	14 (4.2)
Moderate	243 (73.2)
Number of meal intake per day (%)	
1	54 (16.3)
2	162 (48.8)
3	93 (28)
4	18 (5.4)
5 or more	5 (1.5)
Number of snack intake per day (%)	
0	24 (7.2)
1	101 (30.4)
2	108 (32.5)
3	67 (20.2)
4	19 (5.7)
5 or more	13 (3.9)
Fast food intake	
1 to 4 in a month	105 (31.6)
At least 2 to 4 in a week	193 (58.1)
Once or none in a month	34 (10.2)
Consumption of rich food (%)	
Carbs	51 (15.4)
Carbs, sugar	30 (9.0)
Lipids	14 (4.2)
Lipids, carbs	13 (3.9)
Lipids, carbs, sugar	8 (2.4)
Lipids, sugar	1 (0.3)
Proteins	45 (13.6)
Proteins, carbs	47 (13.6)
Proteins, carbs, sugar	47 (14.2)
Proteins, lipids	6 (1.8)
Proteins, lipids, carbs	6 (1.8)
Proteins, lipids, carbs, sugar	19 (5.7)
Proteins, lipids, sugar	63 (19.0)
Proteins, sugar	2 (0.6)
Sugar	5 (1.5)

**Figure 1:**
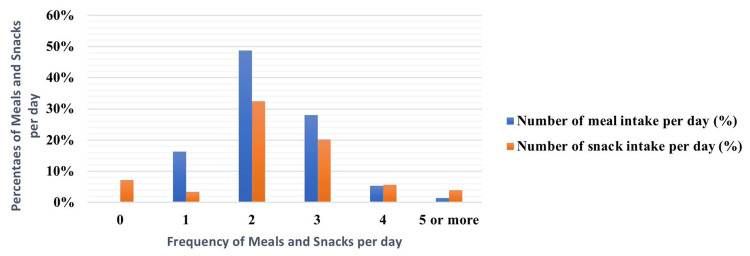
Eating habits of students

The mean number of meals consumed per day was calculated as 2.27 (SD=0.85), whereas the mean number of snacks consumed per day was calculated as 1.98 (SD=1.174) (Figure 1).

**Figure 2:**
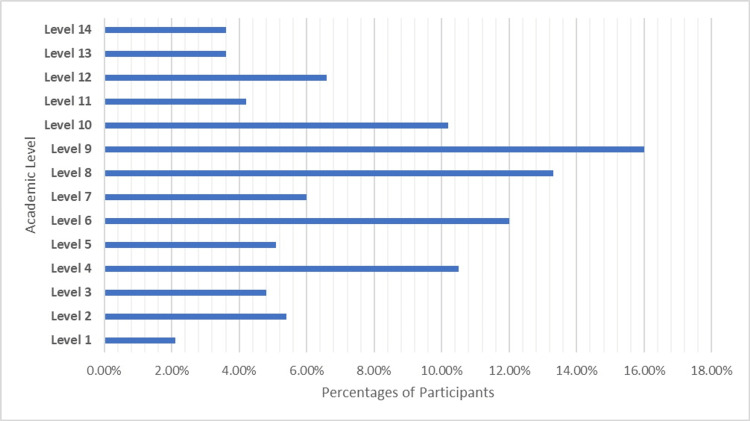
Academic distribution of students

Students were categorized into 14 academic levels: 2.1% (N=7) participants belonged to level 1, 5.4% (N=18) in level 2, 4.8% (N=16) in level 3, 10.5% (N=35) in level 4, 5.1% (N=17) in level 5, 12% (N=40) in level 6, 6% (N=20) in level 7, 13.3% (N=44) in level 8, 16 % (N=53) in level 9, 10.2% (N=34) in level 10, 4.2% (N=14) in level 11, 6.6% (N=22) in level 12, 3.6% (N=12) in level 13, and 3.6% (N=12) in level 14 (Figure 2).

Impact of negative emotions on binge eating

In multiple response options, about 69.27% (N=230) preferred eating during times of stress, aggression, and boredom, and 51.2% (N=170) during loneliness. Regarding binge eating, 19.5% (N=65) reported eating during overwhelming situations, 28% (N=93) revealed during times of great stress, 28.91% (N=96) claimed binge eating when they feel depressed, 6.92% (N=23) students reported during times of disappointment, and 51% (N=170) students shared that loneliness triggers them to eat (Table 3).

**Table 3:** Impact of negative emotions on eating habits and binge eating

Variables		Total (%)
Eating habits	During stress/aggression/boredom	
	Yes	230 (69.27)
	No	102 (30.72)
	During loneliness	
	Yes	170 (51.20)
	No	162 (48.79)
Binge eating	During overwhelming situations	
	Yes	65 (19.57)
	No	267 (80.42)
	During high-stress period	
	Yes	93 (28.01)
	No	239 (71.98)
	During depressed situations	
	Yes	96 (28.91)
	No	236 (71.08)
	During disappointment	
	Yes	23 (6.92)
	No	309 (93.07)

However, a significant association of negative emotions with eating habits was observed in the following ways: boredom (p<0.001*), depression (p<0.013*), a feeling of disappointment (p<0.003*), a feeling of loneliness (p<0.023*), and dissatisfaction toward eaten food (p<0.009*) (Table 4).

**Table 4:** Association between eating habits and relief of negative emotions

Eating Habits	Negative emotions relived with eating						p-value
	Anxiety	Combined disorder	Depression	Disappointment	Stress	Total (%)	
I crave specific food when I am stressed, angry, or bored (stress/aggression and boredom)							
Always	1 (4.3)	22 (25.9)	3 (12.5)	4 (19)	7 (20.6)	45 (13.5)	<0.001*
Never	2 (8.7)	6 (7.1)	1 (4.2)	2 (9.5)	3 (8.8)	45 (13.5)	
Often	7 (30.4)	28 (32.9)	6 (6)	2 (9.5)	8 (23.5)	72 (21.68)	
Rare	7 (30.4)	7 (8.2)	3 (12.5)	3 (14.3)	5 (1.5)	57 (17.16)	
Sometime	6 (26.1)	22 (25.9)	11 (45.8)	10 (47.6)	11 (32.4)	113 (34.03)	
I have problems controlling the amount of certain types of food I eat							
Always	4 (17.)	12 (14.1)	4 (16.7)	5 (23.8)	5 (14.7)	38 (11.44)	0.024*
Never	8 (34.8)	8 (9.4)	3 (12.5)	3 (14.3)	3 (8.8)	56 (16.86)	
Often	4 (17.5)	26 (30.6)	4 (16.7)	5 (23.8)	7 (20.6)	71 (21.38)	
Rare	2 (8.7)	11 (12.9)	4 (16.7)	1 (4.8)	8 (23.5)	62 (18.67)	
Sometime	5 (21.7)	28 (32.9)	9 (37.5)	7 (33.3)	11 (32.4)	105 (31.62)	
I feel that food controls me, rather than I controlling food							
Always	4 (17.4)	12 (14.1)	1 (4.2)	6 (28.6)	6 (17.6)	34 (10.24)	<0.001*
Never	5 (21.7)	11 (12.9)	8 (33.3)	3 (14.3)	11 (32.4)	46 (13.85)	
Often	1 (4.3)	24 (28.2)	6 (25)	5 (23.8)	3 (8.8)	49 (14.75)	
Rare	5 (21.7)	17 (20.0)	3 (12.5)	4 (19)	3 (8.8)	66 (19.87)	
Sometime	8 (34.8)	21 (24.7)	6 (25)	3 (14.3)	11 (32.4)	97 (29.21)	
When I am overwhelmed with things, I have to do							
I eat just as much as usual	4 (17.4)	15 (17.6)	5 (20.8)	5 (23.8)	9 (26.5)	84 (25.30)	0.211
I eat less than usual	11 (47.8)	29 (34.1)	12 (50)	6 (28.6)	9 (26.5)	121 (36.44)	
I eat more than usual	3 (13)	23 (27.1)	3 (12.5)	4 (19)	6 (17.6)	52 (15.66)	
I eat much more than usual	0 (0)	5 (5.9)	1 (4.2)	1 (4.8)	1 (2.9)	13 (3.91)	
I never eat	5 (21.7)	13 (15.3)	3 (12.5)	5 (23.8)	9 (26.5)	62 (18.67)	
During periods of great stress							
I eat just as much as usual	6 (26.1)	7 (8.2)	6 (25)	1 (4.8)	7 (20.6)	63 (18.97)	0.002*
I eat less than usual	8 (34.8)	23 (27.1)	10 (41.7)	9 (42.9)	10 (29.4)	110 (33.13)	
I eat more than usual	4 (17.4)	29 (34.1)	1 (4.2)	4 (19)	7 (20.6)	70 (21.08)	
I eat much more than usual	2 (8.7)	12 (14.1)	3 (12.5)	3 (14.3)	1 (2.9)	23 (6.92)	
I never eat	3 (13)	14 (16.5)	4 (16.7)	4 (19)	9 (26.5)	66 (19.87)	
When I feel depressed							
I eat just as much as usual	5 (21.7)	9 (10.6)	5 (20.8)	4 (19)	6 (17.6)	66 (19.87)	0.013*
I eat less than usual	9 (39.1)	21 (24.1)	10 (41.7)	7 (33.3)	10 (29.4)	105 (31.62)	
I eat more than usual	2 (8.7)	30 (35.3)	5 (20.8)	1 (4.8)	8 (23.5)	71 (21.38)	
I eat much more than usual	1 (4.3)	11 (12.9)	1 (4.2)	4 (19)	4 (2.8)	25 (7.53)	
I never eat	6 (26.1)	14 (16.4)	3 (12.5)	5 (23.8)	7 (20.6)	66 (19.87)	
While preparing for a strenuous task							
I eat just as much as usual	6 (26.11)	23 (27.1)	7 (29.2)	5 (23.8)	7 (20.6)	105 (31.62)	0.123
I eat less than usual	9 (39.1)	28 (32.9)	10 (41.7)	6 (28.6)	14 (41.2)	114 (34.33)	
I eat more than usual	1 (4.3)	13 (15.3)	4 (16.7)	1 (4.8)	3 (8.8)	40 (12.04)	
I eat much more than usual	1 (4.3)	7 (8.2)	0 (0)	3 (14.3)	1 (2.9)	14 (4.21)	
I never eat	6 (26.1)	14 (16.5)	3 (12.5)	6 (28.6)	9 (26.5)	59 (17.77)	
When I am disappointed							
I eat just as much as usual	3 (13)	16 (18.8)	5 (20.8)	2 (9.5)	8 (23.5)	81 (24.39)	0.003*
I eat less than usual	7 (30.4)	21 (24.7)	9 (37.5)	8 (38.1)	12 (35.3)	112 (33.73)	
I eat more than usual	4 (17.4)	20 (23.5)	3 (12.5)	2 (9.5)	4 (11.8)	41 (12.34)	
I eat much more than usual	2 (8.7)	12 (14.1)	1 (4.2)	3 (14.3)	0 (0)	23 (6.92)	
I never eat	7 (30.4)	16 (18.8)	6 (25)	6 (28.6)	10 (29.4)	75 (22.59)	
If time is passing slowly, I look forward to having a snack							
Always	7 (30.4)	18 (21.2)	3 (12.5)	5 (23.8)	6 (17.6)	60 (18.07)	0.187
Never	2 (8.7)	3 (3.5)	0 (0)	1 (4.8)	1 (2.9)	13 (3.91)	
Often	4 (17.4)	30 (35.3)	5 (20.8)	5 (23.8)	13 (38.2)	92 (27.71)	
Rare	2 (8.7)	7 (8.2)	8 (33.3)	1 (4.8)	3 (8.8)	46 (13.85)	
Sometime	8 (34.8)	27 (31.8)	8 (33.3)	9 (42.9)	11 (32.4)	121 (36.44)	
Being alone increases my appetite							
Always	2 (8.7)	18 (21.2)	1 (4.8)	1 (4.8)	4 (11.8)	43 (12.95)	0.023*
Never	5 (21.7)	11 (12.9)	4 (16.7)	7 (33.3)	4 (11.8)	56 (16.86)	
Often	4 (17.4)	22 (25.9)	7 (29.2)	4 (19)	5 (14.7)	60 (18.07)	
Rare	2 (8.7)	9 (10.6)	6 (25)	2 (9.5)	5 (14.7)	63 (18.97)	
Sometime	10 (43.5)	25 (29.4)	6 (25)	7 (33.3)	16 (47.1)	110 (33.13)	
I celebrate with food when I am in a good mood							
Always	9 (39.1)	35 (41.2)	4 (16.7)	6 (28.6)	8 (23.5)	100 (30.12)	0.117
Never	2 (8.7)	0 (0)	1 (4.2)	0 (0)	1 (2.9)	11 (3.31)	
Often	7 (30.4)	27 (31.8)	7 (29.2)	5 (23.8)	8 (23.5)	94 (28.31)	
Rare	0 (0)	6 (7.1)	1 (4.2)	3 (14.3)	1 (2.9)	24 (7.22)	
Sometime	5 (21.7)	17 (20)	11 (45.8)	7 (33.3)	16 (47.1)	103 (31.02)	
If I feel really good, I don’t worry about my diet							
Always	5 (21.7)	18 (21.2)	2 (8.3)	2 (9.5)	8 (23.5)	67 (20.18)	0.469
Never	6 (26.1)	10 (11.8)	2 (8.3)	6 (28.6)	3 (8.8)	41 (12.34)	
Often	6 (26.1)	25 (29.4)	5 (20.8)	3 (14.3)	8 (23.5)	81 (24.39)	
Rare	2 (8.7)	9 (10.6)	5 (20.8)	3 (14.3)	8 (23.5)	61 (18.37)	
Sometime	4 (17.4)	23 (27.1)	10 (41.7)	7 (33.3)	10 (29.4)	96 (28.91)	
When I am happy, having a favorite snack makes me feel even better							
Always	8 (34.8)	34 (40)	8 (33.3)	6 (28.6)	15 (44.1)	119 (35.84)	0.318
Never	1 (4.3)	6 (7.1)	0 (0)	0 (0)	2 (5.9)	17 (5.12)	
Often	7 (30.4)	28 (32.9)	5 (20.8)	3 (14.3)	10 (29.4)	97 (29.21)	
Rare	1 (4.3)	4 (4.7)	2 (8.3)	5 (23.8)	2 (5.9)	28 (8.43)	
Sometime	6 (26.1)	13 (15.3)	9 (37.5)	7 (33.3)	5 (14.7)	71 (21.38)	
I still feel unsatisfied after I eat							
Always	7 (30.4)	7 (8.2)	3 (12.5)	6 (28.6)	3 (8.8)	31 (9.33)	0.009*
Never	3 (13)	12 (14.1)	4 (16.7)	4 (19)	9 (26.5)	73 (21.98)	
Often	3 (13)	17 (20)	2 (8.3)	3 (14.3)	3 (8.8)	50 (15.06)	
Rare	4 (17.4)	20 (23.5)	8 (33.3)	4 (19)	8 (23.5)	81 (24.39)	
Sometime	6 (26.1)	29 (34.1)	7 (29.2)	4 (19)	11 (32.4)	97 (29.21)	

Impact of NELE on weight

The impact of NELE on weight is as follows: stress (p=0.003), depression (p=0.000), disappointment (p=0.001), loneliness (p=0.015), and thought of a strenuous task (p=0.001) (Table 5).

**Table 5:** Impact of NELE on the weight of students

Negative emotions	p-value
Stress	0.003
Depression	0
Disappointment	0.001
Loneliness	0.001
Thought of a strenuous task	0.015

Impact of NELE on BMI

The impact of NELE on BMI is as follows: boredom (p=0.059), feeling of being overwhelmed (p=0.001), depression (p=0.000), thought of a strenuous task (p=0.000), disappointment (p=0.000), stress (p=0.002), and loneliness (p=0.023) (Table 6).

**Table 6:** Impact of NELE on the BMI of students

Negative emotions	p-value
Stress, anger, and boredom	0.059
Overwhelming situation	0.001
High-stress period	0
Depressed situation	0
Thought of a strenuous task	0
Disappointment	0
Feeling of loneliness	0.023

## Discussion

This study aims to investigate the prevalence of binge eating behaviors among medical students, as well as the types of negative emotions that are most strongly associated with binge eating. In this study, the majority of medical students had a BMI within the normal range. This finding is consistent with a study conducted in Saudi Arabia, which reported that only 23% of medical students were overweight, while the majority had a normal BMI [13]. Similarly, just 20% of participants in Nepal were overweight [14]. This study reveals that there is no noteworthy correlation between being a medical student and having a high BMI. Our findings demonstrate that over 50% of medical students possess a moderate socioeconomic status. Conversely, a study conducted in Canada reported that 62.9% of medical students belonged to the high socioeconomic status category [15].

Food consumption and preferences

Our study reveals that approximately half of the medical students surveyed consume only two meals per day. In contrast, a study conducted in Egypt found that over half of the medical students consumed three meals per day [16]. The majority of medical students consume two snacks per day as indicated in our results. Likewise, a study done in India demonstrated that 60.5% of males and 17.5% of females among medical students consume less than three snacks per day [17]. Accordingly, frequent consumption of snacks is not always a usual behavior among medical students. Our results show that more than half of medical students consume fast food two to four times a week. Similarly, a study published in India demonstrated that 98.5% of medical students consume fast food regularly [18]. This clarifies that medical students have bad eating habits due to high consumption of fast food. The majority of medical students consume food rich in protein, lipids, and sugar as shown by our study. In contrast, a study done in Malaysia indicated that 51.5% of medical students had fried food twice or more a week [19]. Variations in the type of food among different studies may be due to the cultural and socioeconomic characteristics of different samples.

Emotional influences on weight and BMI

When under stress, agitated, or bored, more than two-thirds of our subjects chose to eat. It is obvious that medical students' eating habits are influenced by their emotions. This implies that tension, hostility, and boredom rule medical students' lives, or we may attribute these findings to the demanding lifestyle that each medical student leads. It may be inferred from our study's finding that half of the participants eat alone and that students spend the majority of their time alone studying or reading. Pertaining to binge eating, a fifth of the participants in our study admitted to eating during stressful situations, while a third admitted to eating when they were depressed. Half of the individuals said that feeling lonely led them to eat. In our study, depression, the idea of a difficult task, and disappointment were all substantially connected to the detrimental effects of eating on BMI, which were subsequently followed by feelings of overwhelm, stress, and loneliness. The influence of unpleasant emotions associated with eating on weight loss revealed a substantial correlation with depressive symptoms, disappointment, and thoughts of difficult tasks, followed by stress and loneliness. These results support another Saudi Arabian research which also found that individuals tend to lose weight while under stress [13]. These figures contrast with research conducted in Lebanon, which found that weight reduction of more than 20 pounds (9 kilograms) during six months was not connected with any eating disorder [10], demonstrating that the association varies depending on the nation.

Potential implications

The findings of this study highlight the need for further exploration of the relationship between binge eating behaviors, negative emotions, and the socioeconomic and cultural factors specific to medical students. Understanding these associations can have potential implications for the development of targeted interventions and support programs to address and prevent binge eating disorders among medical students. Targeted interventions may include psychoeducation and awareness campaigns, screening and early intervention, counseling and support services, and nutritional guidance. By implementing these targeted interventions, medical schools and institutions can create a supportive environment that addresses the unique challenges faced by medical students, promotes mental well-being, and helps prevent and manage binge eating disorders.

Limitations

One limitation of this study is that the sample size and characteristics may not accurately represent all medical students or the general population. Another limitation is the use of self-reported data, which introduces the possibility of recall and social desirability biases. The cross-sectional design of the study makes it challenging to establish causal relationships, highlighting the need for longitudinal data. Additionally, the self-reported height and weight measurements may have led to misclassification of BMI. It is also important to note that the scale used to measure binge eating behaviors and negative emotions in the present study was not validated. The lack of validation introduces the possibility of measurement error and questions the reliability and accuracy of the results obtained from the scale. Considering these limitations, caution is advised when interpreting the findings, and future research should address these issues for a more comprehensive understanding of the topic.

## Conclusions

In conclusion, the study highlights the prevalence of binge eating behaviors among medical students, as well as the types of negative emotions such as stress, aggression, and boredom, which are significantly associated with binge eating. These negative effects were then followed by feelings of being overwhelmed, stressed, and lonely. Half of the students said that they ate when they felt lonely. Depression, thoughts about difficult tasks, and disappointment were all found to be significantly linked to the detrimental effects of eating on BMI. Students who experience these negative emotions have a significant impact on their ability to lose weight. In other words, negative emotions can be a barrier to weight loss.

Due to the dearth of research on the epidemiology of binge eating in the region, it is often challenging to explain the variations in binge eating prevalence rates, as well as the kinds of negative emotions experienced by medical students living in the Kingdom of Saudi Arabia or the Middle East. Further research is needed to establish the relationship between binge eating and negative emotions, targeting a larger sample size.
